# Reversible Supramolecular Hydrogel for Air-Tolerant Photon Upconversion: Key Development for Photocatalyst Recovery and Product Extraction in Aqueous Medium

**DOI:** 10.1021/acs.chemmater.5c02127

**Published:** 2025-11-05

**Authors:** Paola Domínguez Domínguez, Keita Kuge, Hayato Shoyama, Kiichi Mizukami, Yoichi Sasaki, Sebastian Bonardd, Nobuo Kimizuka, David Díaz Díaz

**Affiliations:** † AFM-NANO, Instituto Universitario de Bio-Orgánica Antonio González (IUBO-AG), 16749 Universidad de La Laguna, Avda. Astrofísico Francisco Sánchez 2, La Laguna 38206, Spain; ‡ Departamento de Química Orgánica, Universidad de La Laguna, Avda. Astrofísico Francisco Sánchez 3, La Laguna 38206, Spain; § Department of Applied Chemistry, Graduate School of Engineering, 12923 Kyushu University, 744 Moto-oka, Nishi-ku, Fukuoka 819-0395, Japan; ∥ 202635 Centro de Física de Materiales (CSIC, UPV/EHU)-Materials Physics Center (MPC), 20018 Donostia-San Sebastián, Spain; ⊥ Department of Polymers and Advanced Materials Physics, Chemistry and Technology University of the Basque Country UPV/EHU, 20018 Donostia-San Sebastian, Spain

## Abstract

The development of upconversion hydrogels that operate in water and under aerobic conditions remains a significant challenge, especially when postreaction separation and component recovery are needed. Here, we present the first supramolecular photocatalytic hydrogel based on a low-molecular-weight gelator (LMWG) that enables green-to-blue triplet–triplet annihilation upconversion (TTA-UC) in aerated water. The hydrogel forms through the coassembly of the LMWG, surfactant, and a sensitizer/emitter pair (PtOEP/DPAS), giving rise to nanostructured compartments that protect long-lived excited triplet states from oxygen quenching. Unlike previous systems based on biopolymers or covalent networks, this supramolecular gelation is fully reversible, allowing the photochemical reaction to be carried out in situ, followed by gel disassembly for the easy extraction of reaction products without the need for heat. Notably, up to 77% of the precious Pt-based sensitizer can be recovered from the gel matrix. This modular, recyclable platform offers an approach to performing photochemical transformations under mild and environmentally friendly conditions, with advantages over previous air-stable UC hydrogels in terms of reusability, extractability, and operational simplicity.

## Graphical abstract

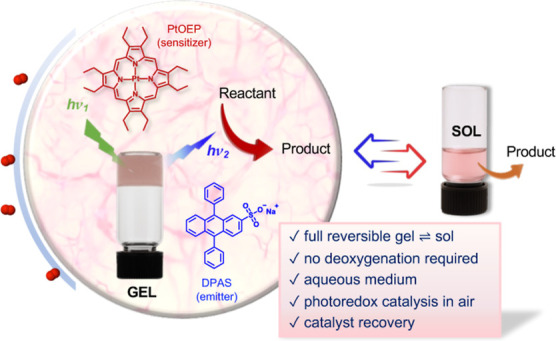

## Introduction

Confinement and compartmentalization are key tools used to achieve efficient and selective reactions.^1,2^ Control of chemical properties and reactivity in a confined reaction environment has been studied in various chemical fields,^3–6^ including photochemistry and photocatalysis.^7–10^ Media such as mesoporous inorganic materials,^11^ microemulsions,^12^ micelles,^13,14^ vesicles,^15^ multilayer polyelectrolyte capsules, proteins and liquid^16^ foams loaded with photocatalysts have been used for confined photoinduced reactions. Among the optical functions studied in these confined spaces and molecular assembly systems, photon upconversion (UC) has recently garnered significant attention as a revolutionary technology that can be utilized for the effective utilization of light.

Photon UC is a nonlinear process that transforms low-energy (longer wavelength) photons into higher-energy (shorter wavelength) photons^17^ and is expected to contribute to the advanced use of sunlight. However, traditional methods, such as second harmonic generation and multistep excitation of lanthanides, require considerable light intensities that are orders of magnitude higher than the solar irradiance and provide low conversion efficiencies even under high excitation intensity.^18–21^ Meanwhile, triplet–triplet annihilation–based upconversion (TTA–UC) uses the collision of two excited triplet states of an acceptor molecule,^17^ which works with low-intensity, noncoherent light. Thus, this provides a promising approach to solving this problem and a clue for effectively overcoming the limits of sunlight-powered devices.

Mechanistically, TTA-UC is a multistep combination of photophysical and photochemical processes that begins with the absorption of low-energy photons (*h*ν_1_) by the donor (sensitizer) in the ground state (*S*_0_) to generate its photoexcited singlet state (*S*_1_), which subsequently produces the triplet excited state (*T*_1_) by intersystem crossing (ISC). The acceptor (emitter) triplets are then populated by triplet energy transfer (TET) from the donor triplets. When two acceptor molecules in their excited triplet states undergo collision during their lifetimes, a higher singlet energy level (*S*_1_) is formed by triplet–triplet annihilation. It consequently generates delayed upconverted fluorescence (*h*ν_2_) to the corresponding ground state (*S*_0_).^19,20,22^ The main drawbacks of TTA-UC are that they have been primarily studied in volatile organic solvents, which are unsuitable for practical applications, and facile quenching of the triplets by dissolved oxygen.^10,23–25^ The use of water, the ultimate green solvent, has been limited by the low water solubility of the sensitizing and emitting dyes.

This solubility challenge can be addressed by solubilizing TTA-UC dyes in the hydrophobic compartment of aqueous molecular assemblies, such as aqueous micelles,^26^ bilayer membranes,^27^ and polymer capsules.^28^ However, it is not easy to protect the excited triplet of TTA-UC dyes from dissolved oxygen using common aqueous self-assemblies, and it is essential to develop molecular systems that are tolerant to oxygen. With respect to practical applications, transparent gel materials are the first candidates for molecular confinement systems that can be developed from a materials chemistry perspective and are expected to show oxygen barrier functionality. TTA-UC in gels formed from covalent polymers and supramolecular polymers has been reported by the groups of Simon, Schmidt and Kimizuka.^8,10,29–33^ Gels containing TTA-UC dyes in the interstitial space of gel nanofibers do not show barrier properties to oxygen.^34,35^ Meanwhile, the solvophobic self-assembly of TTA-UC dyes in hydrogen bond-stabilized gel nanofiber matrixes has shown oxygen barrier properties in polar organic solvents.^8^ Hydrogels that exhibit TTA-UC properties in the presence of dissolved oxygen have been demonstrated by a complex mixture of gelatin, liquid surfactants, and anionic acceptors;^10^ however, to recycle TTA-UC dyes containing polymers such as gelatin may lead to time-consuming thermal separation procedures.^36^ To achieve efficient recovery of expensive triplet sensitizers and products when upconversion is applied to photoreactions, a methodology for creating oxygen-tolerant photoreactor systems needs to be developed based on supramolecular hydrogels. However, design guidelines for these noncovalent polymer hydrogels, which combine operational simplicity, reversibility, catalyst recycling, and oxygen tolerance without requiring biopolymers, are still being developed despite their importance. Addressing this gap would offer opportunities for environmentally friendly photochemistry and light-harvesting photocatalytic systems under ambient conditions.

We herein report the development of a supramolecular hydrogel reactor that exhibits TTA-UC performance in air.^10,24,25,37^ In this work, TTA-UC dyes are solubilized in supramolecular gel nanofibers comprised of liquid surfactants and lipophilic gelators having multiple amide groups capable of forming hydrogen bond networks. This hybrid hydrogel medium reduces the quenching by dissolved oxygen, stabilizing the generated triplet state. A liquid surfactant was used as a component, referring to the previous collagen gel system that yielded a high UC efficiency.^10^ It facilitates the solubilization of TTA-UC dyes in water and is expected to increase fluidity within the hydrogel nanofibers. This work presents a supramolecular reactor design comprising a gelator, a surfactant, and TTA-UC dyes, resulting in the formation of a hydrogel that does not require deoxygenation. The gel system facilitates chemical reaction processes via TTA-UC, enabling efficient recovery of precious platinum-containing triplet sensitizers and extraction of generated products. This paper also describes how molecular self-assembly in multicomponent TTA-UC gels is correlated with their upconversion properties, as well as the application of the supramolecular TTA-UC to the photoreduction of aryl halides. Hydrogels are environmentally friendly materials, so we envisage that they have a wide range of future applications.

## Experimental Section

### Materials

All the solvents and reagents were used as received unless otherwise indicated. Platinum­(II) octaethylporphyrin (PtOEP), Tween 80, rhodamine B, and gelatin from porcine skin (type A) were purchased from Sigma-Aldrich. 9,10-Diphenylanthracene (DPA) was purchased from TCI. *N*-Octadecanoyl-1,5-bis­(l-glutamic acid dibenzyl ester)-l-glutamic diamide (GPD),^38^ and 9,10-diphenylanthracene-2-sulfonate (DPAS)^39,40^ were synthesized according to previously reported methods.

### Preparation of Supramolecular UC Hydrogels

To prepare the chromophore-doped PtOEP/DPAS hydrogel, 3.5 or 8.0 mg of DPAS was mixed with 300 μL of liquid Tween 80 containing PtOEP. These components were mixed entirely by heating, and 12 mg of GPD and 1 mL of an ethanol–water mixture were added to this mixture. The resulting solution was heated to a homogeneous solution and then poured into a cuvette. After cooling slowly to room temperature, the obtained translucent pink gel was incubated overnight before measurement (Figure S1, Supporting Information). The final composition of this hydrogel was [GPD] = 12 mg/mL, [Tween 80] = 0.2 M, [PtOEP] = 68 μM, and [DPAS] = 8 mM or 18.4 mM.

### Confocal Laser Scanning Microscopy (CLSM)

CLSM imaging was performed on a Carl Zeiss LSM 510 confocal microscope. The hydrogel samples were prepared on glass bottom dishes and glass slides (Matsunami Glas Ind., Ltd.). Photoluminescence images were obtained through an LP420 long-pass filter using a diode laser at λ_ex_ = 405 nm for blue fluorescence and through an LP560 long-pass filter using a HeNe laser at λ_ex_ = 543 nm for red fluorescence.

### Characterization and Measurement of the UC of Hydrogels

UV–visible absorption spectra were recorded on a JASCO V-670 spectrophotometer. Time-resolved photoluminescence lifetime measurements were carried out via a HAMAMATSU Quantaurus-Tau compact fluorescence lifetime spectrometer C16381. The excitation and detection wavelengths used for fluorescence lifetime analysis were 405 and 442 nm, respectively. The excitation and detection wavelengths were set at 531 and 646 or 661 nm for phosphorescence lifetime analysis. The TTA-UC emission lifetimes were obtained with the excitation and detection wavelengths set at 531 and 442 nm, respectively.

Photoredox catalytic dehalogenation experiments were conducted using a 532 nm diode laser (200 mW, RGB Photonics) as the excitation source. The doped hydrogel samples were irradiated under ambient air conditions.

For TTA-UC emission measurements, a diode laser (532 nm, 200 mW, RGB Photonics) was used as the excitation source, and the hydrogel samples were tested in an air atmosphere. The laser power was controlled by combining the software (Ltune) and a variable neutral density filter and measured via a PD300-UV photodiode sensor (OPHIR Photonics). The diameter of the laser beam (1/*e* ^2^) was measured at the sample position via a CCD beam profiler SP620 (OPHIR Photonics), and the area was estimated (4.03 × 10^–4^ cm^2^). The emitted light was collimated by an achromatic lens, and the excitation light was removed via a notch filter (532 nm). The emitted light was again focused by an achromatic lens to an optical fiber connected to a multichannel detector MCPD-9800 (Otsuka Electronics). The absolute photoluminescence quantum yields of the dyes in the hydrogels were determined using a Hamamatsu C9920-02G instrument equipped with an integration sphere for sample excitation. The TTA-UC efficiency (η_UC_) can be calculated using eq 1 via the relative method with 68 μM rhodamine B in the hydrogel as a reference. Φ_UC_, *A*_UC_, *I*_UC_, *E*_UC_, and η_UC_ represent the UC quantum yield, absorbance at the excitation wavelength,^41^ excitation light intensity, integrated photoluminescence intensity, and refractive index of the hydrogel system, respectively.^42^ The subscript “std” denotes the parameters of the standard systems, and no refractive index correction was necessary in our case as the same medium was used. To normalize the maximum Φ_UC_ to 100%, the equation was multiplied by a factor of 2.

1 $$\eta_{UC}=2\Phi_{UC}=2\Phi_{std}\left(\frac{1-10^{{-A}_{std}}}{1-10^{{-A}_{UC}}}\right)\left(\frac{I_{std}}{I_{UC}}\right)\left(\frac{E_{UC}}{E_{std}}\right){\left(\frac{n_{uc}}{n_{std}}\right)}^{2}$$

The integrated intensity of the upconverted fluorescence was analyzed in the 400.0–500.0 nm region, whereas that of Rhodamine B was analyzed in the wavelength range of 557.5–800.0 nm. All the measurements were collected under identical experimental conditions.

## Results and Discussion

We employed *N*-octadecanoyl-1,5-bis­(l-glutamic acid dibenzyl ester)-l-glutamic diamide (GPD) (Figure 1a) as a supramolecular gelator for the TTA-UC system. This gelator formed stable gels in water/ethanol (1:1) mixture. The pair of PtOEP/DPAS (Figure 1b,c) was chosen as the donor and acceptor for TTA-UC because it has been employed in previous reports in aqueous media.^10,43–45^ Tween 80, a liquid surfactant, was used to ensure the water solubility of the hydrophobic donors and acceptors (Figure 1d). The liquid surfactant also serves as a coassembling component,^10,36^ which is expected to stabilize hydrophobic GPD gel nanofibers in aqueous ethanol. The presence of these nanofibrous structures was further confirmed by scanning electron microscopy (SEM) imaging (Figure S11, Supporting Information). The samples were obtained first by dissolving the pair of PtOEP/DPAS in Tween 80, followed by the addition of the gelling agent dissolved in water/ethanol (1:1). The mixture was subsequently heated and cooled to form a translucent pink gel (Figure S2b, Supporting Information). UC properties were measured for two different concentrations of the acceptor DPAS (8 mM or 18.4 mM) while keeping the concentration of the donor PtOEP constant (68 μM).

**Figure 1:**
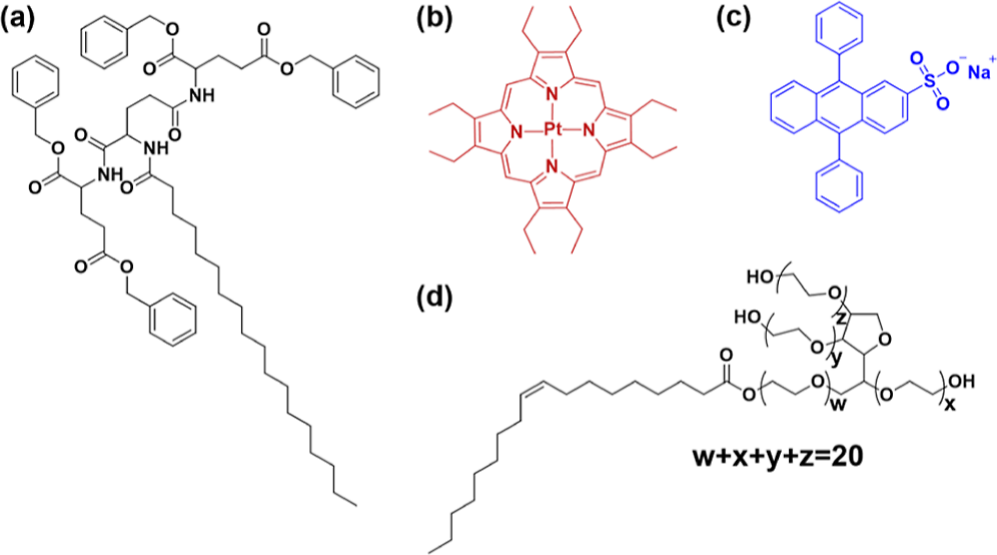
Chemical structures of (a) GPD, (b) PtOEP, (c) DPAS, and (d) Tween 80.

First, confocal laser scanning microscopy (CLSM) was performed to gain insight into the structural features of the GPD/Tween 80 hydrogels and to confirm the colocalization of DPAS and PtOEP. Figure S3 shows an optical microscopy image (a) and a CLSM image (b) of the GPD/Tween 80 hydrogel containing DPAS only. In the CLSM image (excitation at 405 nm with a 420 nm long-pass filter), nanofibrous structures are observed in the area circled by a red square, which shows blue fluorescence of DPAS (Figure S3b). This phenomenon was also confirmed in the superimposed photograph of the two images (Figure S3c).

The above observations indicate that the nanofibers formed a hydrogel and that DPAS was localized within the nanofibers. Although DPAS has a hydrophilic sulfonate group, it is essentially a hydrophobic molecule. Figure S4 shows optical microscopy (a) and CLSM images (b) of the GPD/Tween 80 hydrogel containing only PtOEP. Surprisingly, the red phosphorescence of PtOEP is observed from the round-shaped gel microdots in the air, which is evident from the superimposed image in Figure S4c. As the excited triplet state of PtOEP is readily quenched by dissolved oxygen, phosphorescence is not observed in aerated solutions. The phosphorescence observed in Figure S4b indicates that PtOEP is dissolved in the hydrophobic interior of the GPD/Tween 80 hydrogel and that the gel nanofibers have a barrier property against dissolved oxygen. These CLSM observations indicate that DPAS and PtOEP accumulate inside the gel nanofibers, which is natural considering that the hydrophobic PtOEP is insoluble in the water/ethanol = 1:1 mixture.

The CLSM images obtained for the GPD/Tween 80 gel containing both PtOEP and DPAS are shown in Figure 2. Both blue fluorescence (Figure 2b) and red phosphorescence (Figure 2c) are observed in the gels, indicating that PtOEP and DPAS colocalize in the nanoassemblies in the hydrogel. In addition, CLSM images are homogeneous on the μ-meter scale, and microscopic phase separation between donors and acceptors often observed in bulk crystals^18^ was not detected in the present hydrogel.

**Figure 2:**
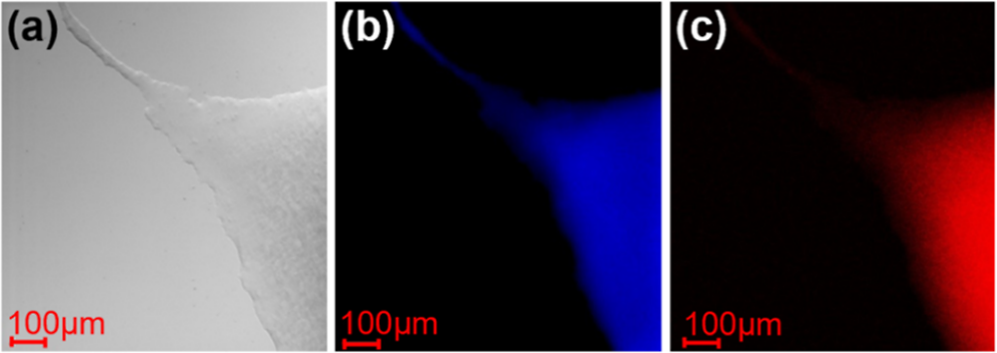
Optical microscope image (a) and CLSM images (b,c) of an edge part of the GPD/Tween 80 hydrogel containing 8 mM DPAS and 68 μM PtOEP. In (b), the CLSM image was obtained by excitation at 405 nm via a 420 nm long-pass filter. In (c), the CLSM image was obtained by excitation at 543 nm using a 560 nm long-pass filter. Scale bars, 100 μm.

We then investigated the TTA-UC characteristics of the GPD/Tween 80 hydrogels containing PtOEP and DPAS (Figures 3a and S5a, Supporting Information). Figures 3b and S5b (Supporting Information) show the luminescence spectra obtained upon excitation of PtOEP with 532 nm light at various incident laser power intensities in the aerated hydrogel (concentrations of DPAS: 8 mM and 18.4 mM, respectively). As expected from the results of the CLSM observations, the pair of PtOEP/DPAS in the GPD/Tween 80 hydrogels showed DPAS emission at approximately 430 nm, indicating that upconversion occurred in the presence of dissolved oxygen. Direct excitation of DPAS in the hydrogels at different concentrations (8 mM or 18.4 mM, λ_ex_ at 405 nm) resulted in similar fluorescence lifetimes of ca. 10.9 and 10.5 ns (Figure S6b). The observed fluorescence spectrum and lifetime of DPAS are similar to those previously reported for gelatin hydrogels containing liquid surfactants,^10^ indicating that the hybrid hydrogel GPD/Tween 80 plays a crucial role in preventing the aggregation of DPAS, which is also supported by the absence of an aggregation-based redshift-component in Figure 3b.

**Figure 3:**
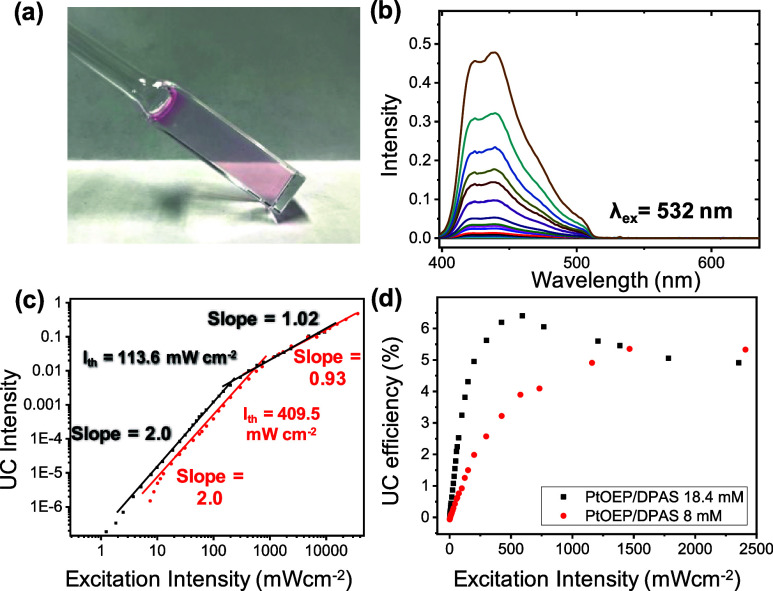
(a) Photograph of a GPD/Tween 80 hydrogel containing 8 mM DPAS and 68 μM PtOEP. (b) Photoluminescence spectra of the air-saturated hydrogel at different excitation intensities with a 532 nm laser at room temperature. [PtOEP] = 68 μM, [DPAS] = 8 mM. A notch filter at 532 nm was used to remove the scattered incident light. (c) Excitation intensity dependence of the UC emission intensity of the air-saturated hydrogel. [PtOEP] = 68 μM, [DPAS] = 8 mM (red) or 18.4 mM (black). (d) UC efficiency η_UC_ of the air-saturated PtOEP-DPAS hydrogels as a function of the excitation intensity at room temperature. [PtOEP] = 68 μM, [DPAS] = 8 mM or 18.4 mM.

In addition, the fluorescence decays of DPAS are unaffected by PtOEP (68 μM), which is noteworthy considering that upconverted singlet energy is usually more or less consumed by undesirable back energy transfer from the acceptor to the donor because of the DPAS fluorescence overlap and PtOEP absorption. The use of large excess acceptors ([DPAS]/[PtOEP] = 118 (DPAS, 8 mM) or 271 (DPAS, 18.4 mM) in the present hydrogel system may have contributed to these favorable TTA-UC characteristics. Moreover, the decays of upconverted fluorescence from DPAS (Figure S6a, λ_ex_ at 531 nm) give millisecond-scale lifetimes. These results indicate that the observed emission is delayed fluorescence via long-lived triplet states. Computational fitting of these curves gave τ_UC_ values (=1/2 τ_T_),^9^ from which triplet lifetimes τ_T_ of 0.26 and 0.36 ms were determined for the hydrogels containing 8 mM DPAS and 18.4 mM DPAS, respectively. These long lifetimes demonstrate that the observed upconversion is based on the triplet-sensitized TTA-UC mechanism.

We note that the TTA-UC emissions are observed in aerated hydrogels. As described previously, the excited triplet states of TTA-UC dyes are quenched by dissolved oxygen when they are located in the interstitial liquid volume of gels, and deaeration is necessary to observe upconversion.^32,33^ Therefore, the observed upconversion under atmospheric conditions confirms that the donors and acceptors in supramolecular hydrogels are localized within the nanostructures self-assembled from GPD and Tween80, which act as a barrier to dissolved molecular oxygen. We presume that the hydrogen bond networks formed among GPD molecules effectively blocked the intrusion of dissolved oxygen.^8,10^

We then investigated the TTA-UC characteristics of these supramolecular hydrogels in more detail. The threshold excitation intensity parameter TTA-UC (*I*_th_), a performance index for the TTA-UC system, was determined using bilogarithmic plots of upconversion emission and excitation intensity shown in Figures 3b and S5 (Supporting Information). The relationship between the incident light intensity and upconversion emission follows a quadratic trend at lower excitation levels, indicating a weak annihilation regime. As the excitation intensity increases, this trend changes to a linear relationship, characteristic of a saturated annihilation regime.^46,47^ The *I*_th_ value marks the critical point where these two regimes intersect, as determined experimentally. From the intersection of the two straight lines (Figure 3c), *I*_th_ values of 409.54 and 113.64 mW cm^–2^ were observed for GPD/Tween 80 hydrogels with DPAS concentrations of 8 mM and 18.4 mM, respectively. The observed smaller *I*_th_ value for the 18.4 mM DPAS hydrogel than for the 8 mM DPAS hydrogel is consistent with the longer triplet lifetime τ_T_ of 0.36 ms, as shown in Figure S6a.

Figure 3d shows the UC efficiencies (η_UC_) determined at various excitation intensities. Since the quantum yield is defined as the ratio of absorbed photons to emitted photons, the theoretical maximum quantum yield (Φ_UC_) of the TTA-UC fluorescence that occurs via the collision of two exited triplets is 50%. However, this value is multiplied by 2 in many reports to obtain a maximum UC efficiency of 100%. As described in the experimental section, this paper normalized the maximum UC efficiency to 100%, denoted as η_UC_ (=2Φ_UC_). The maximum quantum efficiencies η_UC_ observed for GPD/Tween 80 hydrogels containing 8 mM DPAS and 18.4 mM DPAS were 5.4% and 6.4%, respectively. These UC efficiencies are comparable to those of aqueous molecular assemblies (η_UC_ ∼ 7.0%)^48^ but inferior to those of gelatin-containing air-saturated hydrogels (η_UC_ ∼ 10.2–13.5%).^10^

Compared to the gelatin-based system, the UC efficiency of the supramolecular system is lower, likely due to reduced molecular mobility within the highly viscous hydrogen-bonded gel matrix, which, however, plays a critical role in maintaining oxygen tolerance. The triplet energy transfer from the donor to the acceptor and triplet–triplet annihilation processes in solution both require the collision of two molecules, which are limited by slow molecular diffusion in viscous media. In addition, the preferential localization sites of the PtOEP and DPAS molecules in the nanofibrous assemblies of the GPD/Tween 80 gels may differ. Considering that PtOEP is entirely insoluble in aqueous/ethanol media, it would be located deep inside the hydrophobic domain of the GPD/Tween 80 hydrogels. DPAS is also a hydrophobic molecule, as observed in nanofibrous assemblies (CLSM image in Figure S3, Supporting Information); however, its polar sulfonate group has an affinity for water. Therefore, even if colocalized in hydrogel self-assemblies, DPAS would prefer the hydrophilic interface, whereas PtOEP would prefer the hydrophobic interior of self-assembled fibrous nanofibers. Future studies using Förster resonance energy transfer (FRET) or advanced microscopic techniques could help quantitatively assess the precise spatial distribution of both chromophores within the gel nanostructure.

Information on the colocalization, diffusion and collision of PtOEP and DPAS in hydrogels can be obtained by examining the efficiency of the elementary processes of TTA-UC. The following equation describes the quantum efficiency η_UC_ of TTA-UC.^18^

2 $$\eta_{UC}=f\Phi_{ISC}\cdot \Phi_{TET}\cdot \Phi_{TTA}\cdot \Phi_{FL}$$

where Φ_ISC_, Φ_TET_, Φ_TTA_, and Φ_FL_ represent the quantum efficiencies of the donor ISC, TET, TTA, and acceptor fluorescence, respectively. The parameter *f* is the statistical probability of obtaining a singlet excited state after annihilating two triplet states. In the present system, Φ_ISC_ is regarded as 1, and Φ_FL_ was experimentally determined to be 0.89 for DPAS in GPD/Tween 80 gel ([DPAS] = 8 mM) and 0.63 for the PtOEP/DPAS-containing gel ([DPAS] = 8 mM, [PtOEP] = 68 μM). The Φ_TET_ was determined by measuring the change in the phosphorescence intensity of PtOEP in the absence and presence of DPAS (Figure 4). The phosphorescence quantum yield Φ_0_ of PtOEP (68 μM) in the gel determined by the absolute method was 0.221,^42^ whereas in the presence of DPAS (8 mM), Φ decreased to 0.137. Substituting these phosphorescent quantum yields into eq 3 yields an energy transfer efficiency Φ_TET_ of 0.38.

3 $$\Phi_{TET}=1-\Phi /\Phi_{0}$$

**Figure 4:**
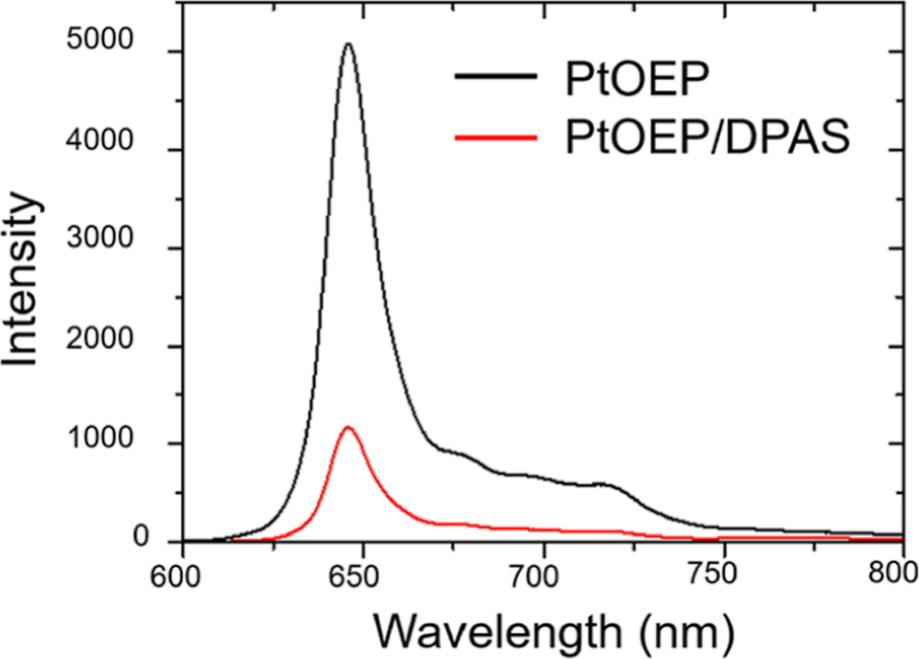
Phosphorescent spectra of PtOEP in aerated GPD/Tween 80 hydrogels. PtOEP (black): [PtOEP] = 68 μM in GPD/Tween 80 gel. PtOEP/DPAS (red): [PtOEP] = 68 μM and [DPAS] = 8 mM in GPD/Tween 80 gel. Excitation wavelength, 532 nm.

The observed phosphorescence of PtOEP in the presence of DPAS suggests the nonuniform spatial distribution of donors and acceptors. There are likely domains where donors are present near acceptors, triplet energy transfer is possible, and phosphorescence quenching occurs. Also, there are donor species where acceptors are not in the vicinity, and phosphorescence emission is preferred over triplet energy transfer. Likely, the fluidity within the GPD/Tween 80 gel fiber is not as low as expected, and it is presumed that interactions between phenyl groups between GPD molecules and the formation of hydrogen bond networks contribute to the increase in microscopic viscosity.

On the other hand, the phosphorescence lifetimes of PtOEP in the GPD/Tween 80 hydrogels are not significantly attenuated compared with those of the gel containing PtOEP and DPAS (Figure S6c, Supporting Information).

The discrepancy between the nondecreased phosphorescence lifetime and the Φ_TET_ of 0.38, as determined by the change in quantum yield, can also be attributed to the nonuniform spatial distribution of donors and acceptors at the nanoscale level. Additionally, the reabsorption of upconverted fluorescence by PtOEP likely occurred due to the high concentrations of chromophores in the hydrogel system. Thus, a detailed discussion of the triplet energy transfer efficiency using the phosphorescence lifetime as an indicator should be avoided in our case.

In the TTA process, singlet, triplet, and quintet multiplicity collision complexes are formed with a statistical probability of 1:3:5. However, the quintet state of polyaromatic organic compounds usually occurs at high energy levels, and the pure statistical factor *f* = 1/9 is expanded to *f* = 2/5.^49^ When the *T*_2_ energy is slightly greater than twice the *T*_1_ energy, the higher energy triplet product *T*_2_ relaxes to the *T*_1_ level,^50^ which has been reported to exceed the statistical factor of 2/5 as in the case of DPA (*f* = 0.52).^49^ The reported *f* values for DPA range from 0.28 ^51^ to 0.52,^50^ depending on the precision of the quantum yield measurement and sample conditions. From the observed η_UC_ = 0.054 ([DPAS] = 8 mM) (Table 1), the product of *f* × Φ_TTA_ in the present hydrogel system is calculated to be 0.16 from eq 2. If we assume Φ_TTA_ to be 1.0, as is commonly done, then an *f* value of 0.16 is obtained. This *f* value is smaller than that in previous reports obtained for deaerated solutions. This will be attributed to underestimated η_UC_ value due to the influence of dissolved oxygen in current supramolecular gels and processes such as concentration quenching of UC fluorescence that are generally unavoidable in high-concentration chromophore systems.

**Table 1:** TTA-UC Efficiencies of the GPD/Tween 80 Hydrogels

donor/acceptor^a^	η_UC’_	Φ_ISC_	Φ_TET_	Φ_FL_	*f* × Φ_TTA_
PtOEP/DPAS	0.054	1	0.380	0.890	0.160

a [PtOEP] = 68 μM, [DPAS] = 8 mM.

The present gelatin-free supramolecular hydrogel is also oxygen tolerant, i.e., donor phosphorescence and upconversion fluorescence are observed in the presence of dissolved oxygen. The ability to effectively block molecular oxygen in noncovalent polymer hydrogels is crucial for applying TTA-UC to photoreaction systems. We then used this system for the first time to perform the photoreduction of aryl halides in an aerated aqueous medium using visible light. Although not the main focus of this work, we preliminarily observed a GC-FID-determined yield of 30% for a model photoreduction reaction carried out under aerobic conditions within the described supramolecular hydrogel using 4′-bromoacetophenone as substrate, DIPEA (11 equiv) as base and Tween 80 as surfactant in a 1:1 mixture H_2_O/EtOH (Figure 5). It is important to highlight that the results was obtained directly without any reaction optimization.

**Figure 5:**
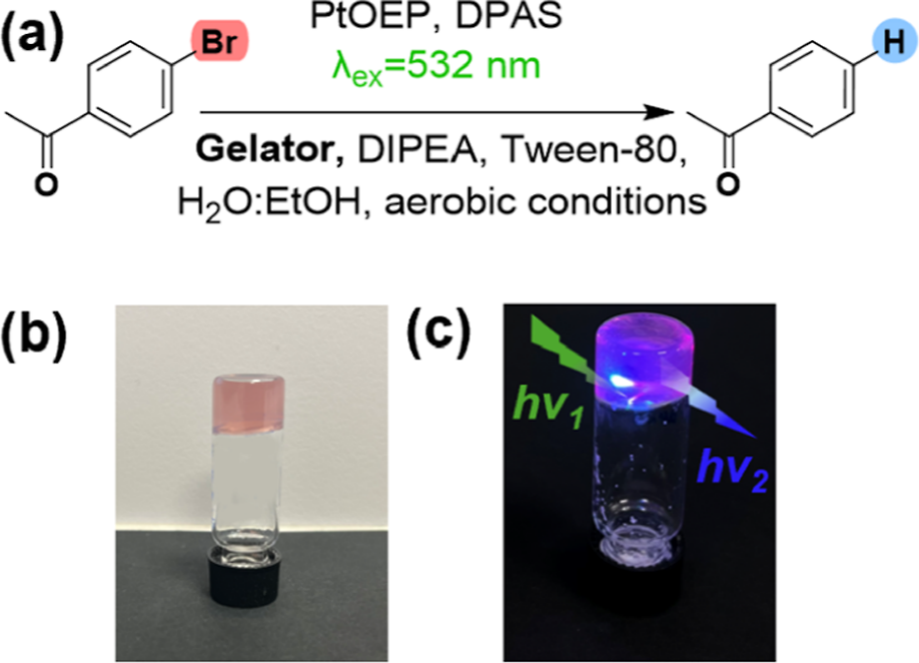
(a) Reaction scheme for the photoreduction of aryl halides. (b) Doped PtOEP-DPAS upconverting hydrogel containing DPAS (18.4 mM), PtOEP (68 μM), 4′-bromoacetophenone (50.2 mM), DIPEA (0.57 mmol), Tween 80 (0.2 M) and GPD (12 mg/mL), in H_2_O/EtOH (1:1, v/v). (c) Doped air-saturated PtOEP-DPAS upconversion hydrogel irradiated at RT for 6 h with a λex = 532 nm laser without any filter.

Notably, a control experiment performed in the neat surfactant (without gel formation) afforded a significantly lower yield under the same conditions, confirming the beneficial role of the hydrogel environment in promoting the photoreduction process (see Supporting Information for details). Previous studies have shown only traces of the desired product in an oxygen atmosphere.^3^ This result highlights the potential of our system for facilitating photochemical transformations in air and aqueous media, and may be further explored for broader substrate scope in future studies. Indeed, other less activated substrates also showed preliminary conversion values between 5 and 21% in air (Scheme S1, Supporting Information).

This hydrogel system offers a significant advantage in that it can be easily destroyed by the use of an organic solvent, such as dichloromethane, which allows for the easy recovery of the encapsulated substrates, providing an efficient and practical method for their release.

To evaluate the efficiency of our system compared with that of other hydrogels previously studied, we performed a comparative study between the hydrogels developed in this work and those made from gelatin. To this end, the recovery of PtOEP from both hydrogels was evaluated (Figure S9), yielding a 77% recovery efficiency in the supramolecular system. In contrast, no significant recovery was achieved with the gelatin-based hydrogel. Bharmoria and co-workers^36^ reported a recovery approach that requires heating to trigger phase separation. In contrast, our method only involves the addition of an organic solvent, offering a notably simpler and more user-friendly alternative. Moreover, the recovery yield in our system (77%) surpasses that reported by Bharmonia and co-workers,^36^ even without prior optimization. While further refinement could enhance this yield, the straightforward disassembly process and the already high recovery efficiency underscore the practical value of our supramolecular system for future recyclable applications. In addition, the photostability of the system was evaluated in air by comparing the emission intensity before and after 6 h of irradiation, revealing that the upconversion emission was well maintained, with only a slight decrease observed after prolonged exposure (Figure S10, Supporting Information).

## Conclusions

In conclusion, we have developed the first supramolecular hydrogel platform capable of performing efficient triplet–triplet annihilation upconversion (TTA-UC) under aerobic aqueous conditions via a low-molecular-weight gelator. The reversible nature of the supramolecular network enables complete disassembly, allowing for the straightforward extraction of photoproducts and the efficient recovery of the photosensitizer.

This level of operational reversibility and component recycling is not feasible with previously reported polymeric or biopolymer-based hydrogels. The system supports green-to-blue TTA-UC emission, sustains photoredox activity in air, and demonstrates high durability and long-lived excited triplet states. Although the current study focuses on single-use performance, future investigations will address long-term stability and the ability to perform multiple TTA-UC cycles without loss of performance. These attributes make this material a compelling platform for sustainable and recyclable photochemical technologies, particularly in fields such as artificial photosynthesis, biocompatible light harvesting, and environmentally benign catalysis.

Ongoing studies are exploring the adaptability of this supramolecular gel platform to other sensitizer/emitter combinations, including those with longer-wavelength excitation and emission profiles.

## Supplementary Material



## Notes

The Supporting Information provides a detailed overview of the hydrogel formation process, including photographs of the materials and microscopy images of GPD/Tween 80 gels prepared with 8 mM DPAS and 68 μM PtOEP. It also features a photograph and emission spectra of the air-saturated GPD/Tween 80 hydrogel containing both PtOEP and DPAS. Additional data include photoluminescence decay profiles, the ^1^H NMR spectrum of GPD, a summary table with the TTA-UC parameters obtained in the study, and the general procedure used for photoredox catalytic dehalogenation in gel under air. Information on PtOEP recovery, calibration curves, and a comparison between the GPD hydrogel and a gelatin-doped system is also included (PDF)

## Abbreviations

CLSM: confocal laser scanning microscopy
DPA: 9,10-diphenylanthracene
DPAS: 9,10-diphenylanthracene-2-sulfonate
GPD: *N*-octadecanoyl-1,5-bis­(l-glutamic acid dibenzyl ester)-l-glutamic diamide
ISC: intersystem crossing
PtOEP: platinum­(II) octaethylporphyrin
TET: triplet energy transfer
TTA-UC: triplet–triplet annihilation-based upconversion
UC: upconversion
η_UC_: TTA-UC efficiency
