# Supplementary material for: Reversible Supramolecular Hydrogel for Air-Tolerant Photon Upconversion: Key Development for Photocatalyst Recovery and Product Extraction in Aqueous Medium
Source: Chem Mater. 2025 Nov 5;37(22):9242–9. doi: 10.1021/acs.chemmater.5c02127 (PMC12661655; doi:10.1021/acs.chemmater.5c02127)
Supplement: Supplementary file 1 [file cm5c02127_si_001.pdf]

Supplementary Information

Reversible Supramolecular Hydrogel for Air-Tolerant Photon Upconversion: Key Development for Photocatalyst Recovery and Product Extraction in Aqueous Medium.

Paola Domínguez Domínguez,[†,‡] Keita Kuge,[#] Hayato Shoyama,[#] Kiichi Mizukami,[#] Yoichi Sasaki,[#] Sebastian Bonardd,[$,&] Nobuo Kimizuka*[,#] and David Díaz Díaz*[,†,‡]

[†]AFM-NANO, Instituto Universitario de Bio-Orgánica Antonio González (IUBO-AG), Universidad de La Laguna, Avda. Astrofísico Francisco Sánchez 2, La Laguna 38206, Spain

[‡]Departamento de Química Orgánica, Universidad de La Laguna, Avda. Astrofísico Francisco Sánchez 3, La Laguna 38206, Spain

[#]Department of Applied Chemistry, Graduate School of Engineering, Kyushu University, 744 Moto-oka, Nishi-ku, Fukuoka 819-0395, Japan

[$]Centro de Física de Materiales (CSIC, UPV/EHU)-Materials Physics Center (MPC) 20018 Donostia-San Sebastián, Spain

[&]Department of Polymers and Advanced Materials Physics, Chemistry and Technology University of the Basque Country UPV/EHU 20018 Donostia-San Sebastian, Spain

Table of Contents

- Figure S1. Hydrogel formation scheme..3

  Figure S2. Photographs of the hydrogels 3
- Figure S3. Optical microscopy image and confocal laser scanning microscopy of GPD/Tween-80 gel, [DPAS] = 8 mM..3
- Figure S4. Optical microscopy image and confocal laser scanning microscopy of GPD/Tween-80 gel, [PtOEP] = 68 μM..4
- Figure S5. A photograph of GPD/Tween-80 hydrogel containing PtOEP and DPAS and photoluminescence spectra of the air-saturated GPD/Tween-80 hydrogel 4
- Figure S6. Emission decay profiles..5
- Figure S7. ^{1}H NMR of GPD..6
- Table S1. Summary table of TTA-UC parameters obtained in this study 6
- General procedure for photoredox catalytic dehalogenation using TTA-UC hydrogel in gel medium and air..7
- Scheme S1. Reaction Scheme for the photoreduction of aryl halides, including other substrates tested..7
- Control experiment using the surfactant as solvent..7
- Recovery of PTOEP from hydrogel system 8
- Figure S8. Calibration data..8
- Figure S9. GPD hydrogel system doped and gelatin doped system.............................9
- Photostability of the hydrogel system 10
- SEM Analysis of the hydrogel system..10

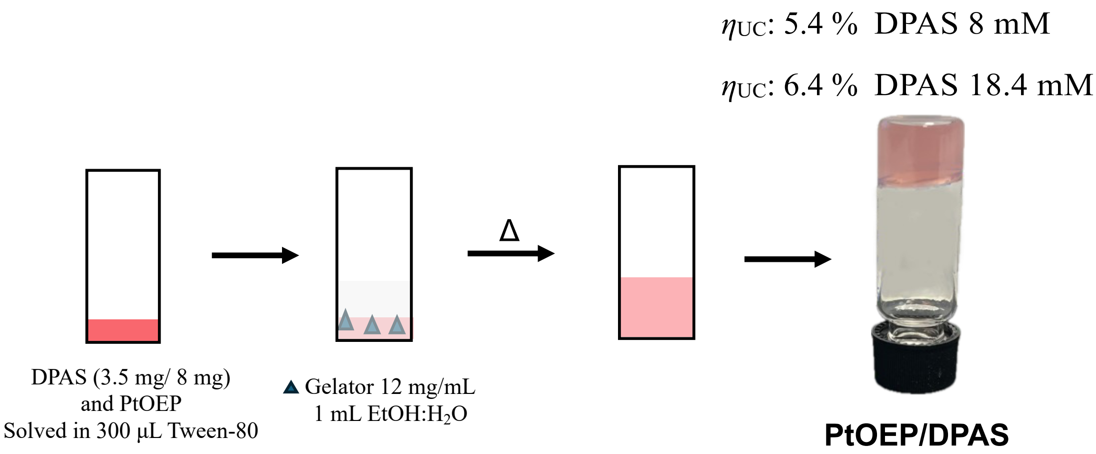

Figure S1. Hydrogels prepared in a 1:1 water:ethanol solvent. The molar ratio is PtOEP:DPAS = 1:270 when using 18.4 mM of DPAS, and PtOEP:DPAS = 1:118 when using 8 mM of DPAS.

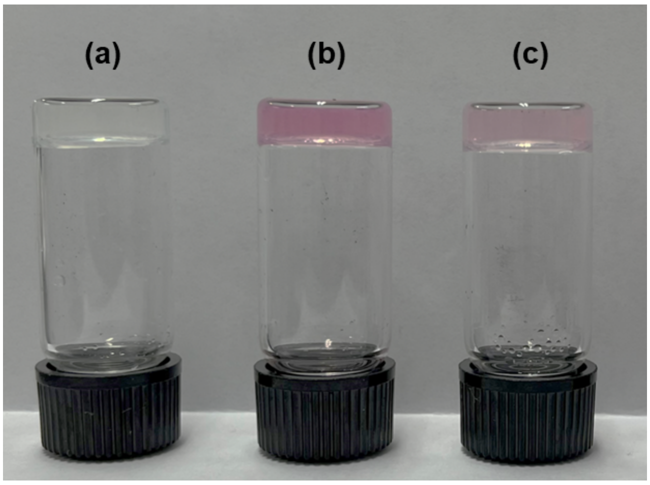

Figure S2. Photographs of (a) DPAS-doped hydrogel, (b) PtOEP-doped hydrogel, and (c) DPAS- and PtOEP-doped hydrogels.

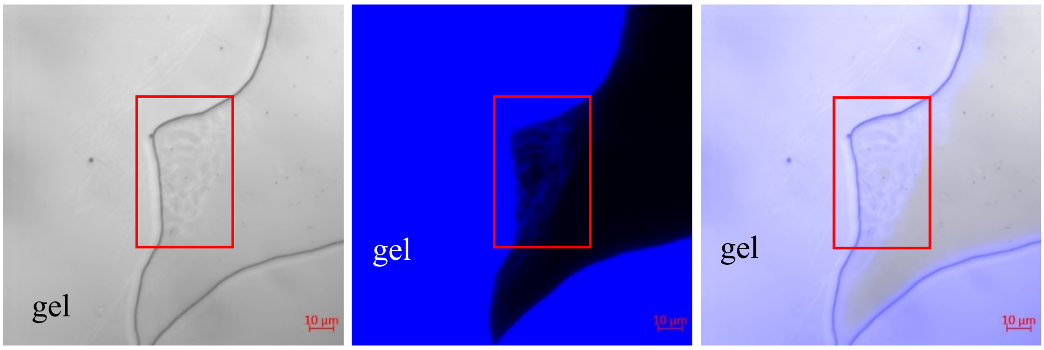

Figure S3. (a) Optical microscopy image of GPD/Tween-80 gel**,** [DPAS] = 8 mM, (b) Confocal laser scanning microscopy (CLSM) image of GPD gel, [DPAS] = 8 mM, CLSM

image was obtained by excitation of the gels at 405 nm using 420 nm long-pass filter. (c) A superimposed view of two images (a) and (b).

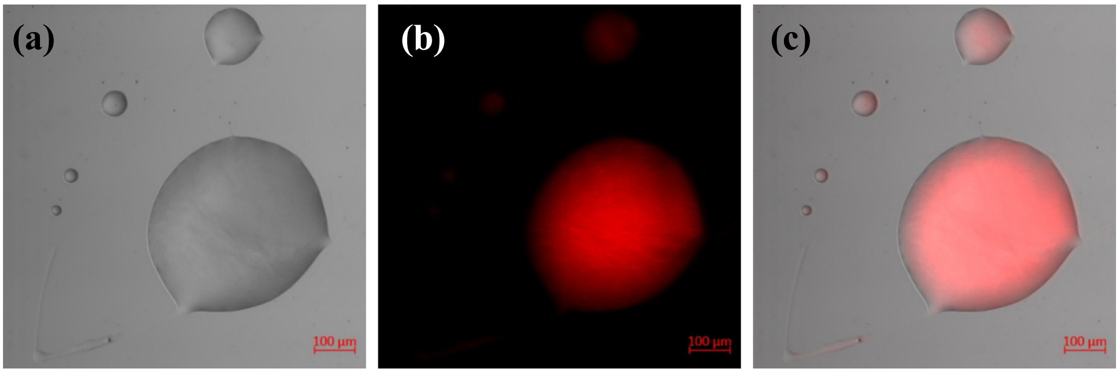

Figure S4. (a) Optical microscopy image of GPD/Tween-80 gel containing 68 μM PtOEP. (b) CLSM images of GPD gel excited by 543 nm with 560 nm long-pass filter. [PtOEP] = 68 μM. (c) A superimposed view of two images (a) and (b).

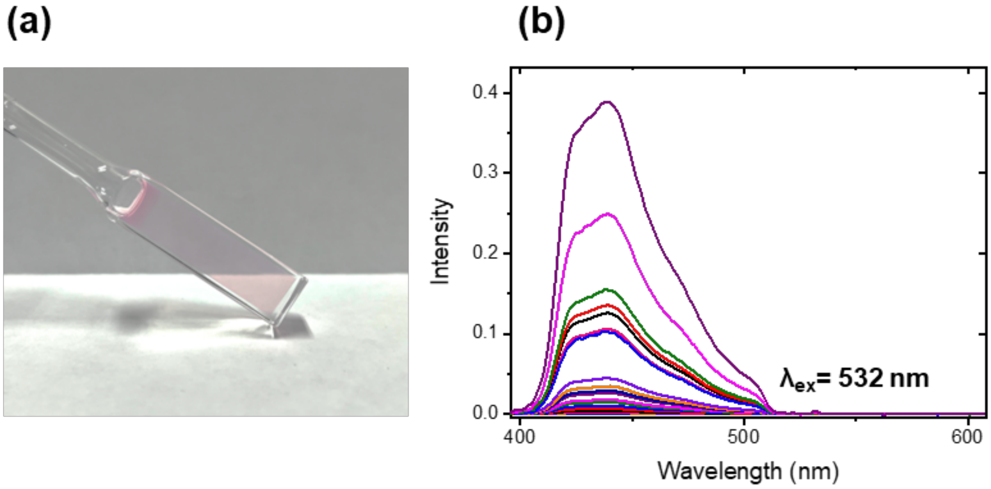

Figure S5. (a) A photograph of GPD/Tween-80 hydrogel containing PtOEP and DPAS. [PtOEP] = 68 μM, [DPAS] = 18.4 mM. (b) Photoluminescence spectra of the air-saturated GPD/Tween-80 hydrogel at different excitation intensities with a 532 nm laser at room temperature. A notch filter at 532 nm was used to remove the scattered incident light. [PtOEP] = 68 μM, [DPAS] = 18.4 mM.

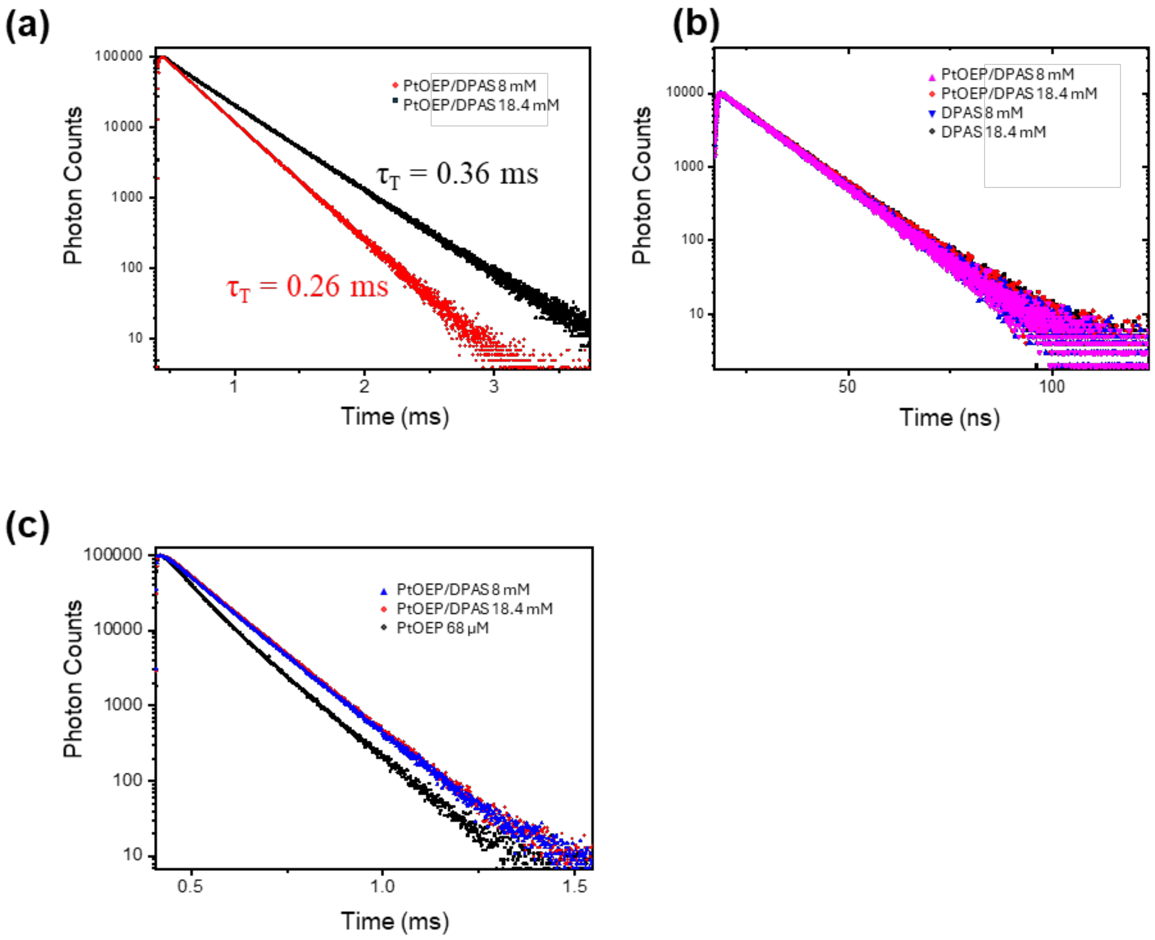

Figure S6. (a) UC emission decay profiles of GPD/Tween-80 hydrogels containing PtOEP/DPAS in air (λ_{ex} = 531 nm, λ_{em} = 442 nm, 510 nm short-pass filter). [PtOEP] = 68 μM, [DPAS] = 8 mM or 18.4 mM. (b) Fluorescence decay profiles of GPD/Tween-80 hydrogels containing PtOEP/DPAS or DPAS in air. [PtOEP] = 68 μM, [DPAS] = 8 mM or 18.4 mM. λ_{ex}= 405 nm, λ_{em} = 442 nm. (c) Phosphorescence decay profiles of GPD/Tween-80 hydrogels containing PtOEP/DPAS or PtOEP in air. λ_{ex} = 531 nm, λ_{em} = 646 nm. [PtOEP] = 68 μM, [DPAS] = 8 mM or 18.4 mM.

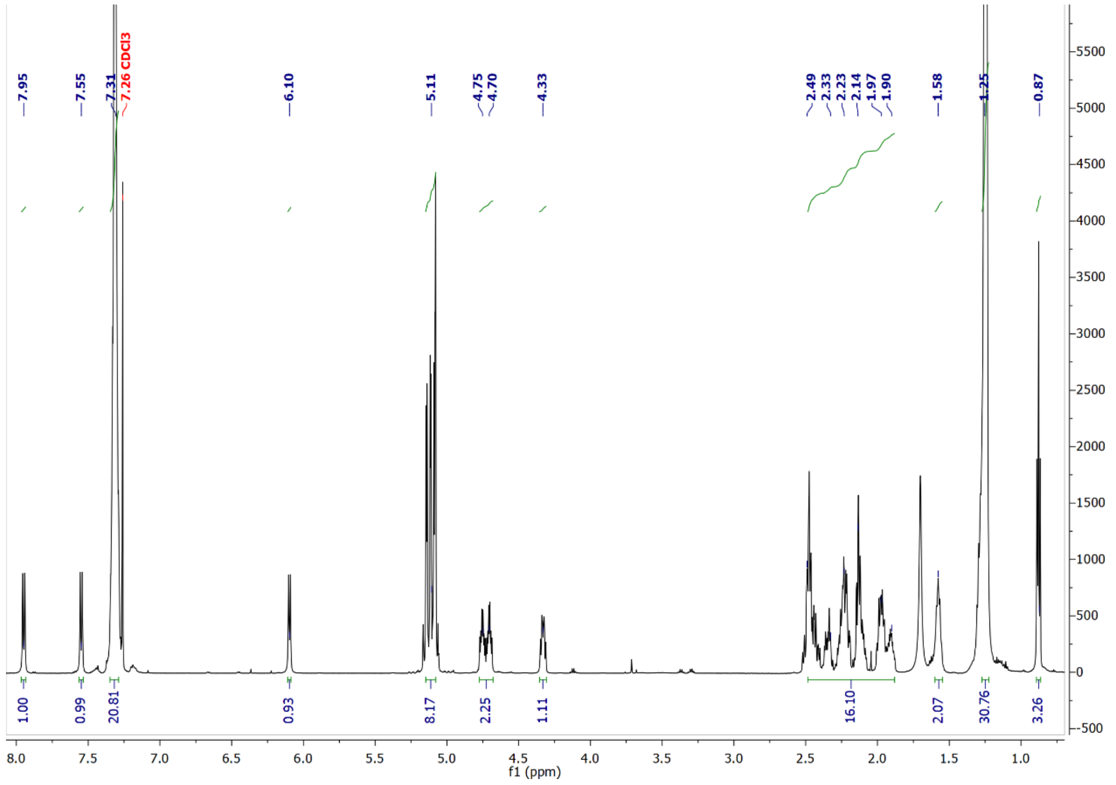

Figure S7. ^{1}H NMR ($CDCl_3$) of GPD. ^{1}H NMR spectrum was recorded on a Bruker AV-500 instrument at room temperature using the residual solvent signal as the internal standard; chemical shifts (δ) are expressed in ppm.

Table 1. Summary Table of TTA-UC Parameters Obtained in This Study.

Donor /68 μM	Acceptor	η_{UC}	I_{th}	$\tau_{A,T}$
PtOEP	DPAS/ 8 mM	5.4%	409.5 $mWcm^{-2}$	0.26 ms (Fig. S5a)
PtOEP	DPAS/ 18.4 mM	6.4%	113.6 $mWcm^{-2}$	0.36 ms (Fig. S5a)

General Procedure for Photoredox Catalytic Dehalogenation Using TTA-UC Hydrogel in Gel Medium and Air.

A 3 mL vial was prepared with the respective aryl halide (0.05 mmol, 1.0 equiv), DIPEA (0.57 mmol, 11 equiv), DPAS (18.4 mM), PtOEP (68 μM), Tween-80 (0.2 M), GPD (12 g/L) and EtOH: H_2O (1:1) (1 mL) and the vial was sealed with a cap (Scheme 1). The reaction mixture was heated with a heat gun until an isotropic solution was obtained. On cooling the mixture to room temperature, a gel was formed. The reaction was irradiated at room temperature using a diode laser (532 nm, 200 mW, RGB Photonics) for 6h, the reaction was monitored using GC-FID. The gel was broken by adding dichloromethane (DCM), distilled water and brine were added for washing. The organic phase was dried over $MgSO_4$, filtered and prepared for injection into a GC-FID using dodecane (10 mM) as an internal standard.

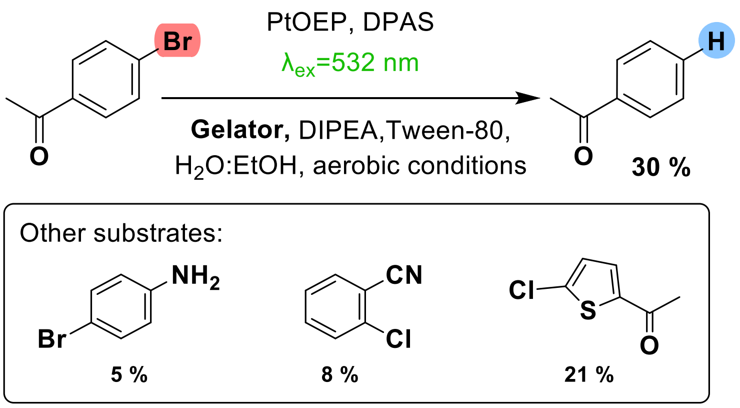

Scheme S1. Preliminary photoreduction of aryl halides under aerobic and aqueous conditions, without reaction optimization. In control experiments lacking the gelator, no conversion or merely trace amounts of product were detected under otherwise identical conditions.

Control experiment using the surfactant as solvent

To evaluate the role of the gel medium, the reaction was also carried out using only the surfactant as solvent, without gel formation. Under these conditions, in a 3 mL vial with the 4'-bromoacetophenone (0.05 mmol, 1.0 equiv), DIPEA (0.57 mmol, 11 equiv),

DPAS (18.4 mM), PtOEP (68 μM) and Tween-80 (0.2 M) the reaction afforded a yield of 12.0 %, confirming that the gel environment plays a significant role in enhancing the reaction efficiency.

Recovery of PTOEP from Hydrogel System

Upon the addition of DCM, rapid dissolution of the hydrogel was observed, allowing easy extraction of PtOEP into the DCM. Flash chromatography was performed using a Biotage Isolera One system to isolate the platinum complex, the most valuable component of the system. Given the small amount of PtOEP in the system, the quantification of the extracted amount is carried out by UV–Vis spectroscopy using a calibration curve (Figure S8).

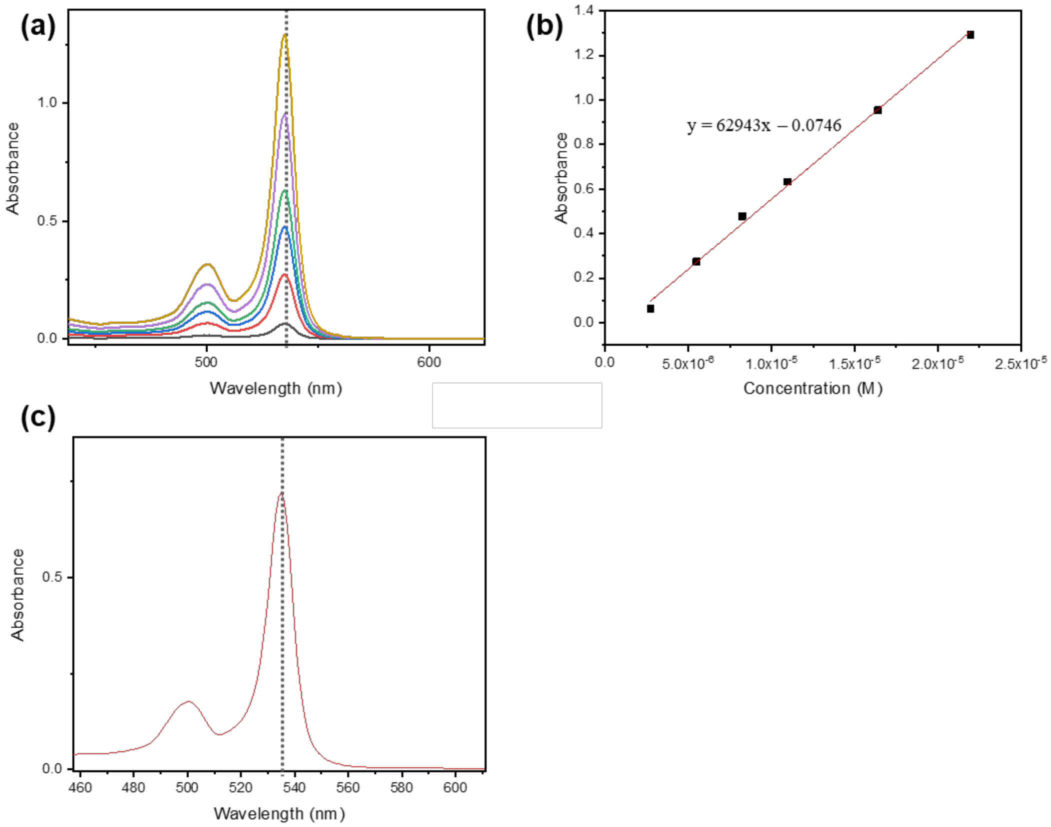

Figure S8. (a) Concentration-dependent absorbances spectrum of PtOEP. The values at 535 nm were used for the calibration. (b) Calibration curve for the quantification of PtOEP. (c) Absorbance spectrum of platinum complex isolated from the system.

On the other hand, a system was carried out in gelatin with the same concentrations, with PtOEP being 1.64 10^{-4} M in a 20% gelatin hydrogel (Figure S9b), we obtained a material that is considerably more resistant to organic solvents, which complicates extraction from the substrate. Even after 24 h in contact with DCM (2 mL), the gelatin remained intact, retaining a large part of the encapsulated PtOEP, which is evident to the naked eye (Figure S9e).

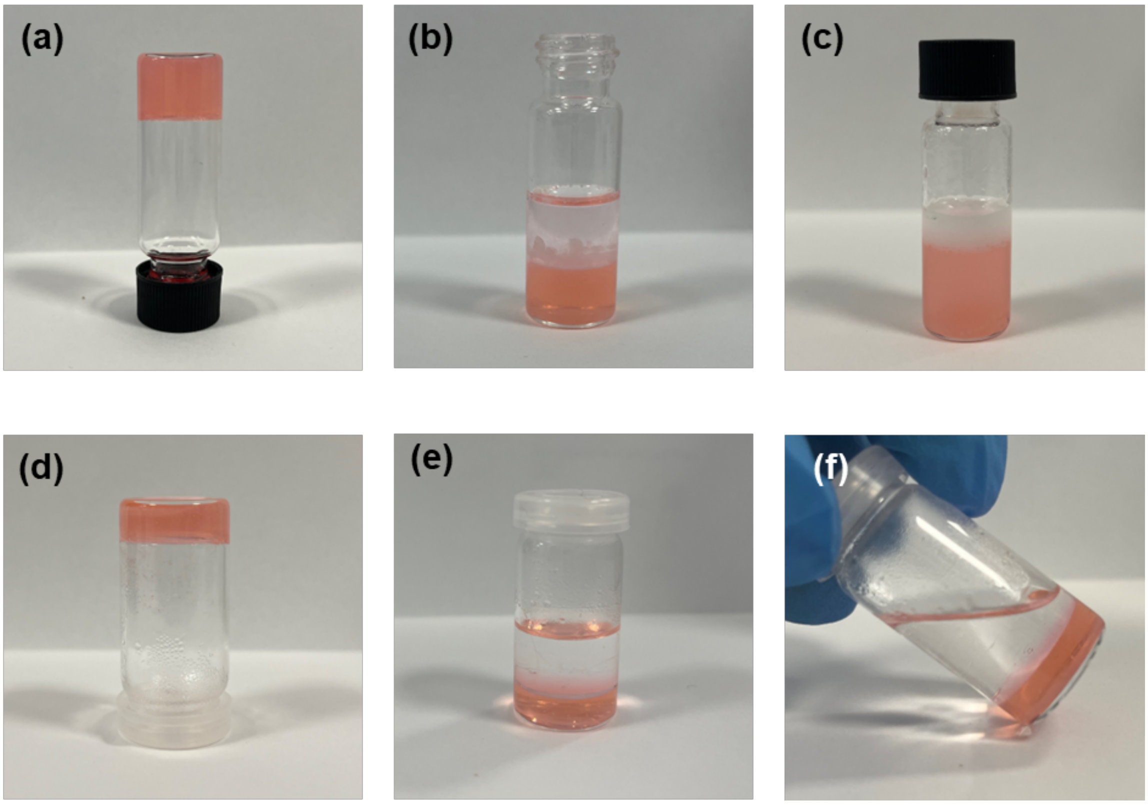

Figure S9. (a) GPD hydrogel system doped with 18.4 mM DPAS, 68 μM PtOEP, and Tween 80. (b) GPD hydrogel system doped with 18.4 mM DPAS, 68 μM PtOEP, and Tween 80 immediately after the addition of DCM. (c) GPD hydrogel system doped with 18.4 mM DPAS, 68 μM PtOEP, and Tween 80 destroyed shortly after the addition of DCM. (d) Gelatin hydrogel system doped with 18.4 mM DPAS, 68 μM PtOEP, and Tween 80. (e) Gelatin hydrogel system doped with 18.4 mM DPAS, 68 μM PtOEP, and Tween 80 immediately after the addition of DCM. (f) The gelatin hydrogel system, doped with 18.4 mM DPAS, 68 μM PtOEP, and Tween 80 in DCM, remains unaltered after being sonicated for 15 minutes.

Photostability of the hydrogel system

The photostability of the hydrogel system was evaluated by measuring its emission intensity before and after 6 h of continuous irradiation under aerated conditions. A slight decrease in intensity was observed (**Figure S10**); however, the change was not significant, indicating that the hydrogel remains largely stable under prolonged irradiation.

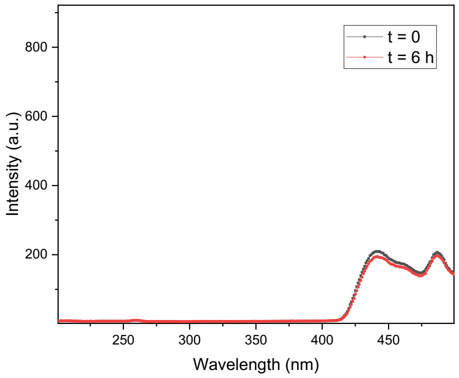

Figure S10. Emission spectra of the hydrogel system recorded before (black line) and after 6 h of continuous irradiation (red line).

SEM analysis of the hydrogel system

To further confirm the formation of nanofibrous structures, the morphology of the hydrogel system was investigated by scanning electron microscopy (SEM). As shown in Figure S11, the SEM images reveal an interconnected network with fibrous features, supporting the presence of nanofiber-like structures within the hydrogel matrix.

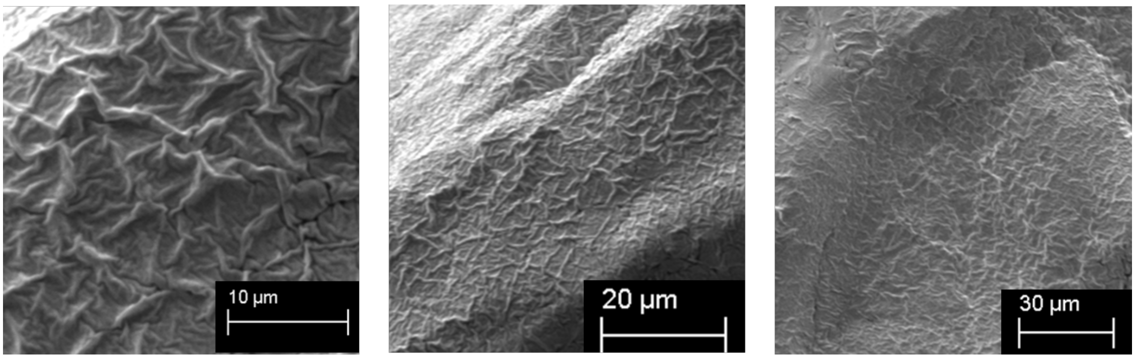

Figure S11. SEM images of the doped hydrogel system at different magnifications.

SEM images of the bulk xerogels were obtained with a ZEISS EVO 15 (resolution 2 nm) equipped with an Oxford X-MAX 50 mm^2 energy-dispersive X-ray (EDX) microanalyzer. Prior to imaging, the samples were frozen at –20 °C for 24 h, followed by solvent removal under high vacuum for 24 hours. SEM images were acquired at the Universidad de La Laguna, within the Servicio General de Apoyo a la Investigación (SEGAI).
